# *Helicobacter pylori* Infection and Extragastric Diseases—A Focus on the Central Nervous System

**DOI:** 10.3390/cells10092191

**Published:** 2021-08-25

**Authors:** Jacek Baj, Alicja Forma, Wojciech Flieger, Izabela Morawska, Adam Michalski, Grzegorz Buszewicz, Elżbieta Sitarz, Piero Portincasa, Gabriella Garruti, Michał Flieger, Grzegorz Teresiński

**Affiliations:** 1 Chair and Department of Anatomy, Medical University of Lublin, Jaczewskiego 4, 20-090 Lublin, Poland; wwoj24@gmail.com; 2 Department of Forensic Medicine, Medical University of Lublin, 20-090 Lublin, Poland; aforma@onet.pl (A.F.); g.buszewicz@umlub.pl (G.B.); michalflieeeger@gamil.com (M.F.); grzegorz.teresinski@umlub.pl (G.T.); 3 Department of Clinical Immunology and Immunotherapy, Medical University of Lublin, 20-093 Lublin, Poland; izabelamorawska19@gmail.com (I.M.); adam.96.michalski@gmail.com (A.M.); 4 Chair and I Department of Psychiatry, Psychotherapy, and Early Intervention, Medical University of Lublin, 20-439 Lublin, Poland; e.sitarz@gmail.com; 5 Clinica Medica “A. Murri”, Department of Biomedical Sciences & Human Oncology, University of Bari Medical School, 70124 Bari, Italy; piero.portincasa@uniba.it; 6 Section of Endocrinology, Department of Emergency and Organ Transplantations, University of Bari “Aldo Moro” Medical School, Piazza G. Cesare 11, 70124 Bari, Italy; gabriella.garruti@uniba.it

**Keywords:** *Helicobacter pylori*, nervous system, gut–brain axis, Parkinson’s disease, Alzheimer’s disease, multiple sclerosis, Guillain–Barré syndrome, Bickerstaff brainstem encephalitis, Devic syndrome, stroke, migraine

## Abstract

*Helicobacter pylori* (*H. pylori*) is most known to cause a wide spectrum of gastrointestinal impairments; however, an increasing number of studies indicates that *H. pylori* infection might be involved in numerous extragastric diseases such as neurological, dermatological, hematologic, ocular, cardiovascular, metabolic, hepatobiliary, or even allergic diseases. In this review, we focused on the nervous system and aimed to summarize the findings regarding *H. pylori* infection and its involvement in the induction/progression of neurological disorders. Neurological impairments induced by *H. pylori* infection are primarily due to impairments in the gut–brain axis (GBA) and to an altered gut microbiota facilitated by *H. pylori* colonization. Currently, regarding a potential relationship between *Helicobacter* infection and neurological disorders, most of the studies are mainly focused on *H. pylori*.

## 1. Introduction

*Helicobacter pylori* (*H. pylori*) is one of the most prevalent pathogens that colonize an estimated 50% of the world’s population [1,2]. Despite the significant *H. pylori* prevalence, the majority of infected individuals remain asymptomatic. This Gram-negative bacterium usually infects the epithelial lining of the stomach and is known to cause a vast array of gastric diseases including, primarily, peptic ulcer disease and gastric carcinoma. Therefore, the eradication of *H. pylori* seems crucial for the prevention of those conditions [1,2,3]. 

*H. pylori* infection constitutes a worldwide issue, although the exact prevalence is strongly associated with the socioeconomic status of the population, with over 80% of adults being infected in developing countries as compared to 20% to 50% in industrialized countries [4]. The exact transmission route of *H. pylori* infection is still largely unknown. Although some sources indicate the possibility of a zoonotic and waterborne transmission of this bacterium, the majority of the infections are thought to be a result of direct, intrafamilial human-to-human transmission, via either oral–oral or fecal–oral routes [1,5,6,7,8,9,10]. As such, improvement of the hygiene and sanitary conditions of the population is one of the most essential ways to decrease the infection rates of *H. pylori* [5].

*H. pylori* is recognized as a principal etiological factor of several gastric diseases, including peptic ulcer disease and gastric carcinoma, as previously mentioned, as well as chronic gastritis or gastric marginal zone/mucosa-associated lymphoid tissue (MALT) lymphoma [1,2,11,12,13]. Though observed less frequently, extragastric manifestations of *H. pylori* infection should also be taken into consideration. *H. pylori* presents the ability to exert its systemic effects via modulation of the gut–brain axis as well as to induce neuroinflammation, reaching the central nervous system (CNS) through the blood, the oral–nasal olfactory route, or gastrointestinal tract (GIT)-associated retrograde axonal transport pathways [14,15]. The effects of *H. pylori* on the gut–brain axis, a bidirectional signaling between the GIT and the brain, can derive from a direct neurotoxic effect, the activation of inflammatory processes in the nerves, and infection-caused microelement deficiencies [14,15,16]. In this review, we aimed to present the current state of knowledge regarding CNS conditions that might be associated with *H. pylori* infection, including Parkinson’s disease (PD), Alzheimer’s disease (AD), multiple sclerosis (MS), Guillain–Barré syndrome (GBS), Bickerstaff brainstem encephalitis (BBE), stroke, migraine, as well as demyelinating diseases such as Devic syndrome [17,18,19,20,21,22,23,24]. 

## 2. *Helicobacter pylori* Characteristics

*H. pylori* is a Gram-negative, microaerophilic, flagellated, helix-shaped bacterium. The bacterium presents a wide spectrum of various adaptation mechanisms which enable its survival in the acidic gastric microenvironment as well as its colonization of the gastrointestinal tract. Crucial for further bacterial colonization, is its ability to form biofilms which, in turn, facilitate bacterial survival and contribute to therapeutic failure. Since 1994, *H. pylori* is recognized as a class I carcinogen related to the onset of gastric cancer, according to the IARC [25]. Even though *H. pylori* colonizes nearly half of the world’s population, the majority of the infected individuals remain asymptomatic and without long-term side effects, e.g., gastritis or peptic ulcer disease. The prevalence of *H. pylori* infection is significantly higher in developing countries as compared to the developed ones, at estimated 85–95% and 30–50% levels, respectively [26]. *H. pylori* still constitutes a major factor responsible for a gastric cancer onset; oncogenic alterations within the gastric mucosa are stimulated by the induction of epithelial–mesenchymal transition (EMT) triggered by bacterial virulence factors [27,28,29]. *H. pylori* pathogenicity depends on the particular strain and so does the genotype and the associated expression of specific virulence factors that facilitate the interplay between the host microenvironment and the bacterium [30]. Table 1 presents major *H. pylori* virulence factors responsible for its pathogenicity. 

## 3. Gut–Brain Axis

The gut–brain axis (GBA) it is a complex network in which the CNS and the enteric nervous system (ENS) interact with each other in a bilateral manner by several mechanisms, including nervous, hormonal, metabolic, and immunological ones [31,32,33,34]. Recently, this relationship has been described as the ‘microbiota–gut–brain axis’ because of the known role of the gut microbiota in maintaining a physiological brain–gut relationship and its participation in the pathogenesis of several diseases [34]. In this complex network, a plethora of interactions take place. The brain—a central, coordinating element of the GBA—receives and releases information via the enteric, sympathetic, and autonomic nervous systems [35,36,37] Further, the hypothalamus–pituitary axis (HPA) as well as sympathetic and cortisol-related immune regulations are involved [38]. The GBA is bidirectional; the CNS takes part in the modulation of ENS functions in several ways—directly and indirectly (directly through changes induced in the microenvironment of the gastrointestinal tract, and indirectly through signaling molecules)—both antagonistically and synergistically [34,38,39]. Three major pathways of GBA communication can be distinguished—the vagus nerve pathway, the neuroendocrine pathway, and the immune-related pathway [31].

It has been proverbially said, that immunity derives from the intestine and this is not an unjustified statement, as the human gut contains the largest concentration of immune cells in the organism [34]. The proper functioning of the intestines appears crucial in guarding autoimmunity, especially due to the fact that the intestines are capable of recognizing and distinguishing potentially harmful bacteria from commensal ones [40]. The latter are involved in both adaptive and innate immunity. The microbiota, through microbe-associated molecular patterns (MAMPs), is involved in promoting the function of cells and cytokines affecting the CNS, which mainly include Il-6, Il-1a, IL-1b, and TNF-α [31].

A vast majority of the gastrointestinal tract functions are controlled by the autonomic nervous system and include bile secretion, motility of the gut, mucosal production, and even the immune response [41]. Normally, in the case of the human body, each action triggers a response; therefore, the information entering the CNS through the autonomic nervous system (ANS) is subsequently transmitted to the organs of the body through closed positive and negative feedback loops [34,42]. The HPA works mainly through the so-called stress hormones and is responsible for the rapid reactions of the body; therefore, disturbances in its functioning exert a significant impact on the entire organism. It seems that in both human and animal models, the HPA is overreactive when the gut microbiota is disturbed, and this overactivity may reversely result in disturbances of the gut microbiota [43,44,45]. The mucosal barrier in the gastrointestinal tract is an extremely important element, constituting the organ’s border and connecting many systems in the human body. It consists of both building and functional elements, including a layer of mucus and phospholipids. Furthermore, the submucosal blood flow has a regulatory effect on the production and release of several mediators. The maintenance of mucosal barrier homeostasis depends on a plethora of bidirectional interacting elements, with a significant role played by the gut–brain axis. As Dolores Sgambato et. al. observed, among the mechanisms included in this cooperation we can find the aforementioned hypothalamus–pituitary–adrenocortical (HPA) system, GABAergic and glutamatergic neurotransmission, thyrotropin release hormone, physiologically active lipids, CGRP, melatonin, as well as peptides such as GLP-1, YY peptide, leptin, and ghrelin. The complexity of this physiology results in a similarly complex pathophysiology: any disturbance in this system can have a negative effect on the integrity of the mucosal barrier [46].

Several microbial molecules are similar to the human ones. Intestinal cells (e.g., enterocytes and secretory cells) are capable of producing and releasing cytokines, chemokines, and, most importantly, endocrine and neurotransmitter molecules (e.g., PYY, GLP-1, 5-HT, GABA) [47,48,49,50]. Furthermore, the microbiota is able to produce metabolites with neuromodulatory properties, with visible results in the ANS [34,51]. Those metabolites include dopamine, 5-HT, GABA, short-chain fatty acids (SCFAs) capable of crossing the brain–blood barrier (BBB), thus influencing neurotransmission within the CNS [31]. Interestingly, several different polymodal receptors are observed within the vagus nerve. The vagus nerve is responsible for gastrointestinal tract innervation and thus it is able not only to recognize physical stimuli like stretching but also to detect the previously mentioned bacteria-produced molecules [52,53]. A study of the so-called ‘cholinergic anti-inflammatory pathway’ proved that the efferent part of the vagus nerve has protective abilities through the inhibition of proinflammatory cytokines [54]. Interestingly, patients who undergo vagotomy because of ulcers appear to be more susceptible to neuropsychiatric diseases [55,56]. On the other hand, stimulation of the vagus nerve in mice increased neurogenesis in the hippocampus [57].

Numerous mechanisms are involved in GBA functions, with remarkable complexity: each element influences the others by creating an intricate network of connections. Even a slight disturbance in one of the many elements can cause a cascade of unexpected reactions, which subsequently might lead to the development of disease. Spichak et al. reviewed over 200 sequencing studies investigating the impact of disturbance of the GBA in the context of neuropsychiatric diseases. After setting exclusion criteria and performing detailed analyses, the scientists found a close link between disturbances of bacterial metabolic pathways and diseases such as Alzheimer’s disease, schizophrenia, anxiety, and depression [58]. Anderson et al. proved a relationship between dysbiosis and multiple sclerosis [59,60]. Ischemic stroke and Parkinson’s disease are also proposed to be related to dysbiosis and, as a result, disturbances within the BBB [36,61].

## 4. Pathophysiology of *Helicobacter pylori* Infection and CNS Diseases

*H. pylori* infection is primarily a recognized etiological factor of gastrointestinal diseases such as gastric ulcer, gastric cancer, acute or chronic gastritis, and functional dyspepsia. Most *H. pylori* infections are asymptomatic and therefore often overlooked; nevertheless, they can have a latent effect on systemic processes in the body. During chronic infection, *H. pylori* becomes a risk factor for the development of MALT lymphoma. Although there have been attempts to link several other infections caused by *Chlamydia psattici*, hepatitis C virus, *Campylobacter jejuni* with the development of MALT lymphoma, it has been indisputably established that the strongest link exists between *H. pylori* gastric infection and MALT gastric lymphoma [62].

Regarding serious consequences of *H. pylori* infection, the so-called “triple therapy” that includes proton pump inhibitors, clarithromycin, and amoxicillin or metronidazole has been proposed. Unfortunately, such therapy may trigger neuropsychiatric symptoms, as well as acute infection by itself. The first review article on the relationship between the psychiatric effects of *H. pylori* therapy and the effects of acute infection was published in 2017 [63]. The data collected in the report suggest that neuropsychiatric symptoms such as dissociation, anxiety, mania, delirium, and psychosis that appear during therapy usually disappear after discontinuing the antibiotics. However, the eradication of *H. pylori* with antibiotics may also have beneficial effects such as the regression of gastric MALT lymphoma in approximately 75% of cases.

The microbiota is composed of about 100 trillion microorganisms that live in the human digestive tract. It creates a natural protective barrier but is also responsible for the secretion of numerous neurotransmitters and neuromodulators, such as serotonin, γ-aminobutyric acid, dopamine, or SCFA including acetate, propionate, and butyrate. During colonization by *H. pylori*, balance in the microbiota is disturbed, which leads to changes in secretion and, consequently, in the homeostasis of the whole organism [63].

Recent findings have revealed that chronic inflammation caused by *H. pylori* infection not only modulates the gastric microenvironment but also may influence other host–pathogen interactions. In 2019, the first immune-histochemical tests of gastric intestinal plexus cells were conducted [64]. The authors assessed plexus adaptive changes in *H. pylori* infection as compared to controls. Inflammation of the ganglia was shown to be associated with the degeneration and loss of neurons. The report showed that *H. pylori*-positive patients revealed a greater density and surface area of the myenteric nervous plexus and a greater number of gastric neuronal cell bodies and glial cells in comparison to the control group.

Since the beginning of the 1990s, many authors have pointed out that *H. pylori* infection affects not only the stomach area, but also other body systems. In 2018, a comprehensive review [65] on this subject was published, that collected data proving that *H. pylori* affects numerous dermatological, neurological, ocular, hematological, cardiovascular, allergic, metabolic, and hepatobiliary functions. The long-term consequences of dysbiosis caused by *H. pylori* infection are significant, especially its influence on the functioning of the nervous system. It has been proven that *H. pylori* infection leads to cognitive decline, dementia, and neurological disorders, which are described in this review.

### Pathophysiology of Helicobacter pylori Infection

The human body has a two-way axis—the brain–gut–microbiota axis—which enables communication between the cognitive and emotional regions of the brain and the functioning of the digestive system [66]. Apart from endocrine and immune pathways, this axis includes the neural one. The HPA axis and the vagus nerve with parasympathetic fibers-produced corticotropin-releasing factor (CRF) play a key role in the communication in this specific network. The fact that, in animal models, bacteria, as a stress factor, can activate the above pathways and induce an anti-inflammatory response through α7-nicotinic acetylcholine receptors (nAChRs) seems to be confirmed [67].

Three possible routes by which *H. pylori* can enter the brain have been identified. The first is the oro–naso–olfactory pathway which enables the penetration of bacteria into the brain through the epithelium in the mouth or the nasopharynx. Another hypothesis assumes that infected monocytes, due to an autophagy defect, can migrate through the BBB, damaged by chronic infection and the production of pro-inflammatory cytokines such as TNF-α. This hypothesis is known as the “Trojan horse theory” and explains the participation of bacteria in *H. pylori*-dependent neuroinflammation, consequently leading to neurodegeneration [14]. Another possible route involves GIT-associated retrograde axonal transport pathways, through which pathogens can also affect the brain [17,68,69,70].

It should be emphasized that *H. pylori* induces pro-inflammatory mechanisms during colonization. The most important factor of virulence is the so-called multifunctional compound VacA, which also plays an important role in the pathogenesis of gastric cancer. Its action on gastric mucosa cells is based on the formation of anion-selective channels, vacuolization, and induction of cellular apoptosis. This, in turn, may affect the functioning of the BBB, as VacA affects bone marrow-derived mast cells (BMD-MCs), resulting in the production of a significant amount of pro-inflammatory cytokines including the interleukins IL-1, IL-6, IL-8, IL-1β, IL-10, IL-12, interferon (IFN) γ, and TNF-α, involved in microgliitis and direct neurotoxicity. TNF-α disrupts the integrity of the BBB by activating matrix metalloproteinases [14]. The protein that induces migration and activation of neutrophils is *H. pylori*-NAP (HP-NAP), which is a pro-inflammatory protein commonly found in individuals with *H. pylori*-related gastritis. Due to a prolonged exposure, the BBB is damaged, and its permeability increases, which induces demyelinating, inflammatory, and edema processes in the CNS. The released inflammatory mediators affect the functions of the hypothalamus and the brainstem by disrupting the neuroendocrine–immune system and activating the HPA axis, which is associated with increased secretion of cortisol and adrenaline [15,71]. It has been proven that *H. pylori* infection can lead to the release of several other neurotransmitters, such as acetylcholine, noradrenaline, dopamine, adrenaline, and serotonin [72].

It should not be forgotten that, in the case of chronic *H. pylori* infection, mucosa atrophy occurs and, consequently, the absorption of vitamin B12 is reduced. It is known that this vitamin exerts a significant influence on the functioning of the nervous system as it produces a neurotrophic and immune-modulating effect in the nervous system. Besides, vitamin B12 is a co-factor in the formation of myelin. B12 hypovitaminosis causes pathological changes in the white and gray matter of the brain, such as sensorimotor polyneuropathy, subacute complex degeneration of the spinal cord, cognitive impairment, and optic neuropathy [73,74]. It is also worth noting that in patients diagnosed with multiple sclerosis, decreased levels of vitamin B12 were detected [75]. This is also a risk factor for cognitive disorders as well as Alzheimer’s disease. A dangerous consequence of B12 avitaminosis may be an indirect increase in the risk of ischemic stroke due to increased levels of homocysteine, whose metabolism involves B12 [76]. The increased level of homocysteine causes an increased number of free radicals and the occurrence of oxidative stress, which is responsible for damage to blood vessels and lipid peroxidation [26].

## 5. Central Nervous System Diseases and *Helicobacter pylori* Infection

### 5.1. Parkinson’s Disease

Parkinson’s disease (PD) is an idiopathic progressive degenerative disease of cells in the substantia nigra causing loss of dopaminergic neurons. The result of degenerative processes is the accumulation of neuronal cytoplasmic inclusions, i.e., Lewy bodies, composed of aggregated alpha-synuclein. In 1996, Altschuler et al. [77] were the first to suggest a relationship between *H. pylori* and PD. Data collected in the first epidemiologic study showed that PD patients (n = 33) had a three-fold higher risk of testing seropositive for *H. pylori* versus controls (n = 78) [78,79,80,81]. The latter cohort studies on groups of many thousands of patients confirmed *H. pylori* infection in 32–70% of patients suffering from PD. In 2017, a meta-analysis by Shen et al. [80] involving 33,125 participants showed that *H. pylori* infection had an adverse effect on disease development. Another research group [82] suggested that *H. pylori* infection causes chronic mucositis, which in turn generates a long-lasting increased secretion of pro-inflammatory cytokines such as TNF-α, IL-1β, and IL-8, damaging the BBB. These processes destroy the brain’s neurons, including dopamine-releasing neurons. An important strategic link between PD and *H. pylori* infection is the toll-like TLR2 receptor. Inflammation in brain cells is associated with TLR2 regulation, which is also important in the function of the intestinal barrier [83]. In another cohort study conducted from 2007 to 2011 on 36 patients diagnosed with PD, it was shown that 50% of them presented IgG antibodies to *H. pylori* [84].

It was observed that the treatment of patients diagnosed with PD may be less effective due to *H. pylori* infection. The reduction of the effectiveness of PD therapy is probably caused by inflammatory changes in the duodenum which damage the mucosa, impairing the absorption of L-3,4-dihydroxyphenylalanine (L-dopa). A poor absorption of the basic drug of PD therapy hinders the course of the treatment [80]. That is why *H. pylori*-positive patients usually require higher doses of drugs and show a better response to treatment after *H. pylori* eradication, which improves their response to L-dopa therapy [84,85].

### 5.2. Alzheimer’s Disease

Alzheimer’s disease (AD) is a neurodegenerative disease characterized by the loss of neurons in the cerebral cortex and subcortical regions. It is the most common cause of dementia leading to death. Many studies emphasize a link between the pathogenesis and development of AD and *H. pylori* infection [72,82,86,87,88,89,90,91]. The authors emphasize the significant increase in the risk of AD development in *H. pylori*-infected individuals, declaring that eliminating the infection may alleviate the symptoms of AD [92,93]. Huang et al. [86] described a 1.6-time higher risk of developing the disease in patients with positive results for the presence of this bacterium in their body. Roubaud Baudron et al. [87] reported a similar risk for developing dementia after 20 years of infection compared to individuals without infection. It should be noted, however, that the association between *H. pylori* and AD is not unequivocal and still requires further research, since some reports question the existence of a statistically significant relationship between *H. pylori* and AD [94]. Immunological studies are more explicit. Significantly higher levels of specific antibodies anti-*H. pylori* IgG in both cerebrospinal fluid (CSF) and serum were detected in AD patients as compared to controls [95]. It should be noted that in the group of patients with antibodies, a more severe course of the disease was observed.

An increased incidence of apolipoprotein E 4 (ApoE4) polymorphism, which is the strongest risk factor for the development of AD, was claimed by others [95]. Other pathogenetic associations of AD with *H. pylori* infection, such as a possible activation of platelet aggregation, oxidative stress, and cross-reaction between *H. pylori* antigens and endothelium, were also considered.

### 5.3. Multiple Sclerosis

Multiple sclerosis (MS) is a chronic immune-related demyelinating disease of the CSN [96]. Although its exact pathogenesis remains unclear, it is hypothesized that, among other things, environmental factors might be involved [97]. It is proposed that bacterial infections may prevent MS outcome, or, in contrast, may be involved in the pathogenesis of the disease [96,98,99]. Research has shown that *H. pylori* infection in MS patients is less common as compared to patients with other neurological diseases [100]. Studies in mice showed a surprisingly beneficial effect of infection on the clinical symptoms of MS [101]. The protective role of infection is probably related to the inhibition of Th1 and Th17 responses.

There are reports on the potential role of *H. pylori* infection in the etiopathogenesis of various autoimmune diseases, including MS [102,103]. Kira et al. described *H. pylori* presence in esophagogastroduodenoscopy in more than 80% of patients with MS and, as compared to a control group with iron-deficiency anemia, those results were statistically higher [96]. The main histopathological finding in patients with MS and co-existing *H. pylori* infection was atrophic gastritis and, interestingly, those patients presented with other autoimmune-related disorders such as ulcerative colitis [21,96,104]. In another study based on patients diagnosed with MS and neuromyelitis optica (NMO), 82.1% NMO patients and 73.8% MS patients were seropositive to *H. pylori*. However, seropositivity was statistically higher only in the group of NMO patients [105]. When it comes to clinically isolated syndrome (CIS), which may suggest the possibility of MS occurrence, a Greek population of patients diagnosed with CIS tended to have a higher prevalence of *H. pylori* infection [106]. A discovery of higher levels of anti-*H. pylori* heat shock protein 60 (HSP60) in patients diagnosed with MS might support the hypothesis of a pathogenetic role of *H. pylori* infection in MS [107]. These higher levels correlated positively with Expanded Disability Status Scale (EDSS) and duration of illness, especially in secondary progressive multiple sclerosis (SPMS), and were proposed to be used as disease progression biomarkers [107]. Heat shock proteins (HSPs) are present in both prokaryotic and eukaryotic organisms as one of the most evolutionary conserved proteins with possible immunogenic properties [108]. In a healthy organism, the own HSPs do not promote an immunological response; however, there are some data about their involvement in autoimmunity. On the other hand, prokaryotic HSPs are engaged in immune responses, and some of them are proven to be a trigger of autoimmune diseases, such as Guillain–Barre syndrome or myalgic encephalitis [109,110]. Interestingly, both prokaryotic and eukaryotic HSPs share some epitopes, and, consequently, this may promote cross-reactivity. Nevertheless, this topic still remains unclear [108]. There is also an observation of the overexpression of HSPs of the HSP70 family in MS patients’ brains. Furthermore, the overexpression of HSP70-related genes and of genes of the immune system was also reported, so it is suspected that this protein may be involved in the pathogenesis of MS [111].

Mainly, when it comes to the protective role of infections in preventing the development of autoimmune diseases, we rely on the so-called hygienic hypothesis. The protective role of *H. pylori* infection in MS has been investigated in meta-analyses. Of 82 identified records, only 9 were included, so the result 1553 cases of MS and 1553 healthy controls were described [112]. In this meta-analysis, there was a statistically lower prevalence of *H. pylori* infection in the group of patients diagnosed with MS as compared with healthy individuals [112]. Likewise, in a Japanese study based on 105 patients with MS, seropositivity against *H. pylori* was significantly lower than in healthy volunteers. Furthermore, in the group of patients with consecutive MS, there was an inverse correlation with the EDSS score [100]. Another research described lower EDDS in seropositive women as compared to seronegative ones. In males, interestingly, this statistic was the opposite [113]. Moreover, in the study conducted by Mohebi, patients with MS and coexisting *H. pylori* infection had a lower range of neurological complications [114]. Similar observations were described in several studies: *H. pylori* seropositivity was found to be less frequent in patients with MS [114,115,116]. Pedrini et al. described differences in seropositivity for *H. pylori* infection between MS patients and healthy volunteers as statistically significant, with a decrease being discovered in a female population, but not in a male one [113]. Interestingly, in three independent experiments performed on mice with experimental autoimmune encephalitis (EAE)—an experimental model of MS—*H. pylori* infection statistically reduced the severity of EAE and lowered the number of pathogenic T lymphocytes within the central nervous system [101]. Additionally, seropositivity for *H. pylori* was also assessed in a group of MS patients and was significantly lower than in a group of healthy individuals. This experimental study strongly supports the hypothesis of a preventive role of *H. pylori* infection in MS [101]. The proposed mechanism of protection by *H. pylori* infection in EAE and MS involves restoring the balance between Th1, Th17, and Treg lymphocytes subsets via several pathways, especially through those connected to IL-10 functions and CCR6–CCL20 interaction [117,118,119,120,121,122].

Despite the relatively high interest in the subject, it remains to be established whether, in the context of MS, infection with *H. pylori* could act as a protective or harmful factor. It should be remembered that most of the studies presented in this review were typically conducted on small groups of patients recruited from one specific ethnic population. The incidence of *H. pylori* infection as well as the incidence of MS vary depending on age, ethnicity, socioeconomic status, and gender. More studies are needed on this topic.

### 5.4. Guillain–Barré Syndrome

Guillain–Barré syndrome (GBS) is a potential life-threatening immune-mediated disorder with ongoing demyelization within peripheral nerves, typically triggered by infections [18,120]. GBS is mainly triggered by *C. jejuni* and *M. pneumoniae*, as well as by common viral infections—Epstein–Barr virus (EBV), Cytomegalovirus (CMV), hepatitis E virus, or Zika virus [23,123,124,125,126,127]. GBS is an acute autoimmune neuropathy; the disease manifests itself as a progressive paralysis of the extremities of the distal-proximal pattern. The disease can be life-threatening if the respiratory muscles or the autonomic nervous system are paralyzed. The most commonly recognized form of the disease is acute inflammatory demyelinating polyneuropathy (AIDP). Besides, there are some clinical variants of the disease. One of the variants that was described first is Miller Fisher syndrome (MFS) [128]. MFS is a less common form of GBS, with at least two symptoms among reflection, ophthalmoplegia, and ataxia. There are also variants that weaken the respiratory system such as BBE [129]. Both patients with MFS and BBE develop anti-GQ1b antibodies. Rare cases have been described of Bickerstaff’s encephalitis associated with *M. pneumoniae* infection [130]; however, the pathophysiology of the disease following *M. pneumoniae* infection is largely unknown.

Epidemiological studies confirmed the occurrence of this variant in up to 10% of GBS cases. Some variants of GBS, such as acute motor axonal neuropathy (AMAN) and acute motor and sensory axonal neuropathy (AMSAN), are associated with *C. jejuni* infection, which is the most common cause of bacterial gastroenteritis [131]. It was found that the infection worsened the course of the disease and was not associated with a good prognosis. The pathogenesis of GBS and its variants is not fully understood. It is currently believed that GBS originates from autoallergic neuritis, which is mediated by T lymphocytes against peripheral nerve myelin proteins and antibodies to myelin glycolipids. These antibodies are detected in the serum of GBS patients [132].

It was not initially assumed that *H. pylori* infection would cause acute demyelinating diseases, but after epidemiological studies, a significantly increased number of *H. pylori* infections was observed in patients with GBS compared to individuals without the disease. A relationship between *H. pylori* infection and GBS is also considered due to the fact that IgG antibodies against *H. pylori* have been detected in the serum and CSF of GBS patients [82,133,134]. Specific IgG antibodies against VacA of *H. pylori* have been also found in the CSF of GBS patients [135]. The authors assumed that the mechanism that explains the influence of *H. pylori* infection on the pathogenesis and course of GBS is related to molecular mimicry between peripheral nerve gangliosides, in particular the sialic acid component, and *H. pylori* antigens. In patients diagnosed with MFS, the presence of anti-VacA antibodies in the cerebrospinal fluid was also detected. The authors observed a similarity between the sequences of the antibodies and ion transport proteins in the membranes, which is the likely cause of interference in Ranvier nodes [24].

Molecular mimicry is proposed as the potential mechanism triggering the disease outcomes. Over the recent years, *H. pylori* has been proposed as a potential pathogen involved in the immunoethiopathogenesis of this disease, although this hypothesis has not been proven yet. The meta-analysis performed by Dardiotis et. al., based on six case–control studies, proved that both in the serum and in the CSF of patients there was a higher level of anti-*H. pylori* IgG antibodies, as compared to the control group [23]. These results may indicate the association between *H. pylori* infection and GBS pathogenesis. Test performed on the CSF of patients diagnosed with GBS revealed the presence of anti-VacA IgG antibodies [136]. Interestingly, there are molecular similarities between human ATP and VacA, which may result in VacA binding to Schwann cells and lead to demyelization [135]. Interestingly, high serum levels of anti-*H. pylori* IgG were associated with a worse clinical status and demyelination within the proximal parts of peripheral nerves [133].

### 5.5. Bickerstaff Brainstem Encephalitis

In 1957, Bickerstaff described cases with a unique presentation of ophthalmoplegia, ataxia, altered consciousness, and hyperreflexia. The neurological symptoms were preceded by infection and typically ended with patients spontaneously recovering, similarly to the recoveries observed in GBS and MFS patients. Although the three diseases were at first thought to be separate conditions, Bickerstaff himself pointed out their resemblance [137]. In 1992, a study led by Chiba discovered an anti-GH1b antibody in patients with MFS, which was quickly followed by reports of those antibodies being present in BBE patients as well [138,139]. As a result, a more inclusive nomenclature was coined when referring to the overlap between BBE and MFS, i.e., ‘anti-GH1b antibody syndrome’, although cases of BBE and MFS with the presence of anti-GM1b and anti-GalNAc-GD1a, but not anti-GH1b, antibodies were similarly identified [140,141].

Although most cases of BBE are preceded by *C. jejuni* and *H. influenzae* infection (26% and 8% of cases, respectively, according to Ito et al.), recent findings, although scarce, seem to indicate the possibility of *H. pylori*-triggered BBE [24,142]. *H. pylori* has been proven to be able to induce immune responses that may trigger and perpetuate a damage to the nerves, as observed in several neurodegenerative disorders, including GBS [133,143]. This conclusion is further supported by the results of the meta-analysis performed by Dardiotis et al., in which a connection between *H. pylori* infection and GBS was established [23]. As a result, we can speculate that, due to the similarities in pathophysiology of GBS and BBE, BBE can be triggered by that pathogen in a similar way [24].

### 5.6. Devic Syndrome

Neuromyelitis optica (NMO)/Devic syndrome is an inflammatory, demyelinating disease of the CNS characterized by severe attacks of optic neuritis and myelitis [144]. It is commonly divided into two types: a relapsing NMO, associated with autoimmunity, and a monophasic NMO, of which 88% is related to a preceding infection [145,146]. Another factor associated with NMO is the presence of anti-AQP4 antibodies, which can be detected in 60–90% of patients diagnosed with this condition [147]. As *H. pylori* is more commonly found in anti-AQP4 antibody-positive patients compared to anti-AQP4 antibody-negative ones, we can speculate that *H. pylori*-caused chronic infection may contribute to the development of NMO via molecular mimicry between bacterial AQP and human AQP4 [148,149].

### 5.7. Stroke

In 2012, Wang et al. [150], based on a meta-analysis, showed that chronic *H. pylori* infection and the presence of CagA-positive strains appeared to be risk factors for ischemic stroke. The authors suggested that *H. pylori* infection increased the expression of a number of inflammatory mediators and activated platelets and factors involved in coagulation [82]. In view of emerging clinical reports on a positive predictive value of *H. pylori* infection for stroke, in 2013 [151], the results of an extensive cohort study on almost 1000 patients were published. However, the study did not confirm the hypothesis that *H. pylori* infection would increase the risk of stroke. The possible mechanisms of such influence were not identified. Some authors suggest however, the possible affinity of on *H. pylori* for the atherosclerotic plaques [152].

### 5.8. Migraine Headaches

There is no evidence that *H. pylori* infection is more common in patients suffering from migraine. However, there are few clinical studies that point to the effectiveness of antibiotic therapy in the treatment of migraine [153,154].

## 6. Conclusions

Beside various gastrointestinal impairments such as peptic ulcer disease, MALT lymphoma, and adenocarcinoma, *H. pylori* infection has been reported to be associated with other extragastric diseases, amongst which neurological disorders where thoroughly discussed in this article. Frequent *H. pylori* infection leads to significant alterations in the composition of the gastrointestinal microbiome, the production of free radicals, changes in neuropeptide expression, as well as both axonal and neuronal damage that might lead to the induction of neurological impairments or alter the outcome of already existing ones e.g., due to the exacerbation of symptoms. The gut–brain axis plays a crucial role in infection and further clinical outcomes. It should be taken into consideration that any alterations in the gut microbiota (e.g., due to *H. pylori* infection) could have a significant impact on other systems of the organism. So far, on the basis of a thorough review of the currently available literature, we assume that *H. pylori* infection might be linked to such neurological disorders/impairments as PD, AD, MS, GB, BBE, Devic syndrome, or even stroke. Even though there are several studies published regarding a possible link between the *H. pylori* infection and neurological disorders, the literature is still scarce, and this matter requires further investigation and proper evaluation. It is also worthwhile mentioning that *H. pylori* is one of the most widely described species, while the other species of *Helicobacter* have hardly been studied.

## Figures and Tables

**Table 1:** *Helicobacter pylori* virulence factors that facilitate bacterial survival, colonization, and carcinogenesis.

Virulence Factors	
Urease	Flagellum
Cytotoxin-associated gene A	Vacuolating cytotoxin A
Catalase	Superoxidase dismutase
Lewis antigens	Arginase
Phospholipases	Lipopolysaccharide
Blood group antigen-binding adhesin	Sialic acid-binding adhesin
Outer inflammatory protein A	Duodenal ulcer promoting gene A
Adherence-associated lipoprotein A and B	LacdiNAc-specific adhesin
*Helicobacter pylori* outer membrane protein Q	*Helicobacter pylori* outer membrane protein Z
Induced by contact with epithelium gene A	Cholesteryl α-glucosyltransferase
γ-glutamyl-transpeptidase	Neutrophil-activating protein
High temperature requirement A	Heat shock proteins

## Abbreviations

AD	Alzheimer’s disease
AIDP	Acute inflammatory demyelinating polyneuropathy
AMAN	Acute motor axonal neuropathy
AMSAN	Acute motor and sensory axonal neuropathy
ANS	Autonomic nervous system
ApoE4	Apolioprotein E4
BBB	Brain-blood-barrier
BBE	Bickerstaff brainstem encephalitis
BMD-MCs	Bone marrow-derived mast cells
CIS	Clinically isolated syndrome
CMV	Cytomegalovirus
CNS	Central nervous system
CRF	Corticotropin releasing factor
CSF	Cerebrospinal fluid
EAE	Experimental autoimmune encephalitis
EBV	Epstein–Barr virus
EDSS	Expanded Disability Status Scale
ENS	Enteric nervous system
GBA	Gut–brain axis
GBS	Guillain-Barré syndrome
HPA	Hypothalamus–pituitary axis
*H. pylori*	*Helicobacter pylori*
HSP60	Anti- *H. pylori* heat shock protein 60
HSPs	Heat shock proteins
L-dopa	L-3,4-dihydroxyphenylalanine
MALT-lymphoma	Marginal zone/mucosa associated lymphoid tissue lymphoma
MAMPs	Microbial-associated molecular patterns
MFS	Miller Fisher syndrome
MS	Multiple sclerosis
nAChRs	α7-Nicotinic acetylcholine receptors
NMO	Neuromyelitis optica
PD	Parkinson’s disease
SCFAs	Short-chain fatty acids
SPMS	Secondary progressive multiple sclerosis
